# Exploring men’s pregnancy-planning behaviour and fertility knowledge:a survey among fathers in Sweden

**DOI:** 10.1080/03009734.2017.1316531

**Published:** 2017-05-04

**Authors:** Maja Bodin, Lisa Käll, Tanja Tydén, Jenny Stern, Jennifer Drevin, Margareta Larsson

**Affiliations:** a Department of Women’s and Children’s Health, Uppsala University, Uppsala, Sweden;; b Centre for Gender Research, Uppsala University, Humanistiskt Centrum, Uppsala, Sweden;; c Department of Health Promotion, Sophiahemmet University, Stockholm, Sweden;; d Department of Public Health and Caring Sciences, Uppsala University, Uppsala, Sweden

**Keywords:** Fathers, fertility knowledge, gender equality, lifestyle, preconception health, pregnancy planning

## Abstract

**Introduction:**

Research about pregnancy-planning behaviour mostly focuses on women, even though pregnancy planning usually also concerns men. The purpose of this study was to investigate how men plan for family, and to measure their fertility knowledge after having become fathers.

**Material and methods:**

Data were collected in 2014 as part of a Swedish longitudinal pregnancy-planning study. Men were recruited through their female partner one year after childbirth. Participants were asked to fill out a questionnaire about pregnancy planning, lifestyles, and fertility.

**Results:**

Of the 796 participants, 646 (81%) stated that the pregnancy had been very or fairly planned, and 17% (*n* = 128) had made a lifestyle adjustment before pregnancy to improve health and fertility. The most common adjustments were to reduce/quit the consumption of alcohol, cigarettes, or snuff, and to exercise more. First-time fathers and those who had used assisted reproductive technology to become pregnant were more likely to have made an adjustment. Fertility knowledge varied greatly. Men with university education had better fertility knowledge than men without university education.

**Conclusion:**

Our findings indicate that there is variation in how men plan and prepare for pregnancy. Most men did not adjust their lifestyle to improve health and fertility, while some made several changes. Both pregnancy-planning behaviour and fertility knowledge seem to be related to level of education and mode of conception. To gain deeper understanding of behaviour and underlying factors, more research is needed.

## Introduction

For many years, public health scholars have been advocating that pregnancies should be carefully planned, mainly because of the associations found between unplanned pregnancies and adverse health outcomes (see for example Shah et al. (1)). Lately attention has been given to the negative impact of unhealthy lifestyles prior to conception, and women in many countries are now recommended by health care institutions for example to stop smoking and drinking alcohol, to eat more healthy food, and to take folic acid supplements, already before pregnancy is confirmed (2,3).

Pregnancy-planning interventions usually focus on women’s bodies and doings, even though pregnancy planning usually also concerns men. There is, however, a growing body of evidence indicating that sperm quality and quantity can be negatively affected by factors such as obesity, smoking, and exposure to endocrine disrupting agents and persistent organic pollutants (4). Male fertility is also affected by age, and advanced age has been associated with longer time to pregnancy (5) as well as psychiatric and academic morbidity of the child (6,7). It is, however, important to note that postponement of parenthood can also have benefits, e.g. increased maturity, and social and cultural capital, which can suppress the negative effects.

At the same time, recent literature demonstrates that a significant proportion of men have low knowledge about fertility issues. Many men overestimate the success rate of fertility treatment (8,9), are unaware of at what age there is a marked decline in women’s fertility (10–12), and are ignorant of the relationship between men’s age and reproductive outcomes (13). Women on the other hand tend to be more aware of specific pre-pregnancy health behaviours and initiate more preconception health conversations with their partners (14).

The levels of engagement in family-planning issues are influenced by gender-specific expectations on procreation and the general notion of women as being more interested in reproductive matters than men are. In Sweden, as in many other countries, family planning is often regarded as a female interest and responsibility (15). Intersected with gender, there are also norms related to for example age (e.g. timing of parenthood) and sexuality (e.g. heteronormativity) that influence people’s awareness and behaviour with regard to procreation (16). Marsiglio et al. argue that men’s perception of procreative responsibility is based on their so-called ‘procreative consciousness’ (17). The procreative consciousness depends on factors like fecundity perception, emotional response, knowledge, temporality, visualizations of the future child, and partner’s impact. The process by which men become aware of their procreative potential is relevant to their behaviour, and men who develop a greater depth of knowledge usually have a richer and fuller procreative consciousness. Marsiglio et al. conclude from their interviews that many men do not reflect upon their sperm in any particular way until they become aware that they have impregnated someone or experience infertility.

Even though men’s fertility awareness has been evaluated in several studies, there is still a lack of knowledge concerning men’s pregnancy-planning behaviour. For example, we do not know if men are aware of, and influenced by, the health and lifestyle discourse surrounding fertility and pregnancy. Furthermore, most available studies on men’s fertility awareness and family-planning intentions are based on samples of well-educated men or men with infertility problems. This limits our knowledge to a group of men with higher socio-economic position and/or with experiences that most likely have raised their awareness. Our study aims to broaden this picture by exploring the ways in which men plan for pregnancy, and to measure their fertility knowledge after having become fathers. The aim was further to explore if there were any differences in behaviour and knowledge between groups with different socio-demographic characteristics and reproductive histories.

## Material and methods

### Setting

Data collection was carried out in Sweden, and the Swedish context is in itself of relevance to the outcome. Sweden is a welfare state where gender equality and involved fatherhood have become a norm and a social and political ideal. Governmental initiatives encourage the gender-equal dual-earner/dual-carer model by generous access to parental leave and childcare. However, when it comes to public equality initiatives, little attention has been given to the time before pregnancy, i.e. the preconception period. There are for example no national guidelines for preconception health care for men (3).

### Study design

The study is based on cross-sectional data retracted from a longitudinal Swedish pregnancy-planning study. The longitudinal study initially involved antenatal clinics in nine counties in middle and northern Sweden. All 196 antenatal clinics in the counties were invited, and 144 clinics accepted participation. From another county council an additional 19 antenatal clinics with high percentages of immigrant women were invited, of which nine accepted participation. The clinics were located in both urban and rural areas. Midwives working at the antenatal clinics were asked to recruit pregnant women at their first antenatal visit. The aim was to recruit 5,000 women, which would represent about 4.5% of all women who give birth in Sweden yearly. Potential participants were given oral and written information about the study. Participation was voluntary, and participants could withdraw at any time. The recruitment process has previously been described in detail by Stern et al. (18).

**Figure 1.**
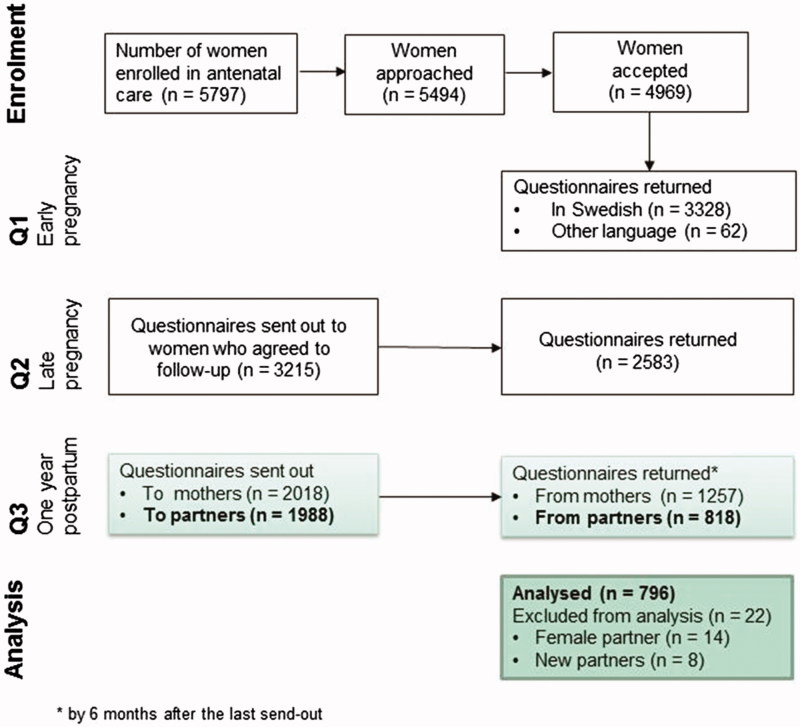
Flow chart of the study procedure.

The study procedure is illustrated in Figure 1. Women were asked to fill out questionnaires in early pregnancy (Q1), late pregnancy (Q2), and one year post partum (Q3). Partners were recruited post partum. An invitation letter and a questionnaire to the partner were added in the envelope containing the woman’s Q3. The partner questionnaire was only sent to those women who had confirmed being in a relationship in early pregnancy, and it was only available in Swedish. If the questionnaire was not returned by three weeks, a reminder was sent by text message, e-mail, or mail to the birth mother, as no contact details to the partner were available. The partner was informed that if s/he filled out and returned the questionnaire, s/he simultaneously gave consent to participation. In total 1,987 partner questionnaires were sent out, and 818 (41%) were returned. Twenty-two questionnaires were excluded from this study since the partner was either a woman (*n = *14) or a new partner (*n = *8).

### Instruments

The questionnaire contained 64 main questions, of which some had follow-up questions and some were measurement instruments with a number of items. Twenty-six questions were explored in this specific study. These were: 10 items about background characteristics, 11 concerning family planning, and five assessing fertility knowledge. Most items were multiple-choice questions. Estimations of current health status, level of pregnancy planning, and attitude towards planning were measured on five-point Likert scales.

Lifestyle adjustments were measured by the question ‘Did you do anything to improve your health and fertility in preparation for pregnancy?’ The respondent could choose from 15 predetermined answers, including ‘I did not do any of the above *before* pregnancy’, and/or describe in free text the adjustments made. Fertility knowledge was measured by five questions with open answers, but with a given unit (days/per cent/years). Questions 1 and 3–5 have been used in previous studies in the Swedish setting (8,19,20). Question 2 was developed for this study. To assess fertility knowledge, a template was developed from available literature and in discussion with experienced clinicians. Knowledge questions and assessment template are found in Table 1.

**Table 1.** Assessment template to evaluate fertility knowledge.

Question	Underestimated	Correct	Overestimated
1. How long is the ovum viable for fertilization?	<0.5 day	0.5–1 day	>1 day
2. How long does sperm usually survive in the uterus/fallopian tubes?	<2 days	2–3 days	>3 days
3. What is the probability that a 25-year-old woman becomes pregnant if she has unprotected intercourse with a young man at ovulation?	<20%	20%–30%	>30%
4. At what age begins a marked decline in a woman’s ability to conceive?	<33 years	33–37 years	>37 years
5. What are the chances of having a child through IVF, for each attempt?	<20%	20%–30%	>30%

### Statistical analysis

Data were analysed using statistical software IBM SPSS Statistics (version 24). The statistical analysis aimed at comparing if there were any differences in background characteristics (independent variables) between fathers who had made a lifestyle adjustment and those who had not (dependent variable). Chi-square test was used for comparison at categorical levels. The categorical variables were parenting experience (first-time father/previous children), mode of conception (spontaneous/assisted), education (dichotomized into Low education = up to high school, and Higher education = university), country of birth (dichotomized into Sweden and Other), and level of pregnancy planning (categorized into three groups: Very/fairly planned, Neither planned nor unplanned, and Very/fairly unplanned). Independent *t* test was used to analyse difference in mean age (years) between fathers who had made a lifestyle change and fathers who had not. Difference in time to pregnancy (TTP) was analysed by Mann–Whitney *U* test since TTP was measured on an ordinal level.

Since we had not asked fathers with previous children how they planned or behaved before previous pregnancies, we also ran the comparative tests with first-time fathers and experienced fathers separately.

Fertility knowledge is presented descriptively by means, median, and range. Chi-square test was used to investigate differences between the three knowledge groups (underestimated, correct, and overestimated) and background characteristics. Missing data were ignored and not imputed since the response rate was more than 95% for each question and hence considered as missing at random. Statistical significance was considered at a *P* value of <0.05.

The study was approved by the regional ethical review board. Questionnaires were coded, personal information was kept separate in a safe place, and only the project co-ordinators had access to the data and the code key. Prior to the study, a pilot study was conducted, and items were adjusted according to the reviews of researchers, clinicians, and laypeople. The knowledge questions were added after the pilot study.

## Results

The study includes 796 men aged 15–57 years (Table 2). Half of the men were first-time fathers. One out of four men (*n = *207) was on parental leave, full or part time, one year after childbirth. The educational levels ranged from no education up to doctoral degree. Most men (92%, *n = *731) were born in Sweden. Cohabiting without being married was the most common type of relationship status. Two-thirds of the fathers (*n = *519) were in good health according to their own estimate. About 12% (*n = *92) were obese, 3% (*n = *24) were daily smokers, and 27% (*n = *210) used snuff (smokeless tobacco) every day.

**Table 2.** Background characteristics of participants.

Characteristics	Frequency, *n* (%)	Mean ± SD
Age (years)	*n = *790	34.3 ± 5.6
Occupation, full or part time^a^	*n = *796	
Permanent job	557 (70.1)	
Self-employed	51 (6.4)	
Temporary position	29 (3.6)	
On parental leave/house dad	207 (26.1)	
Studying	18 (2.3)	
On sick leave/pension	7 (0.9)	
Unemployed	7 (0.9)	
Education (highest completed)	*n = *794	
None	3 (0.4)	
Elementary school	31 (3.9)	
High school	322 (40.6)	
Vocational training	90 (11.3)	
University <3 years	39 (4.9)	
University 3–5 years	285 (35.9)	
PhD degree	24 (3.0)	
Country of birth	*n = *791	
Sweden	731 (92.4)	
Other Nordic country	11 (1.4)	
Other European (non-Nordic)	22 (2.8)	
Outside Europe	26 (3.3)	
Don’t know	1 (0.1)	
Civil status	*n = *792	
Married	319 (40.3)	
Cohabiting	462 (57.9)	
Not living together	11 (1.4)	
Estimated health status	*n = *781	
Very/fairly good	519 (66.4)	
Neither good nor poor	195 (25.0)	
Very/fairly poor	67 (8.6)	
BMI	*n = *787	
<18.5 (underweight)	7 (0.9)	
18.5–24.9 (normal weight)	346 (44.0)	
25.0–29.9 (overweight)	342 (43.4)	
>30 (obese)	92 (11.7)	
Smoking	*n = *787	
Yes, daily	24 (3.0)	
Yes, but not daily	33 (4.3)	
Quit in connection to pregnancy	20 (2.5)	
Quit earlier in life	170 (21.7)	
Have never smoked	539 (68.5)	
Snuff (smokeless tobacco) use	*n = *788	
Yes, daily	210 (26.6)	
Yes, but not daily	18 (2.3)	
Quit in connection to pregnancy	22 (2.8)	
Quit earlier in life	130 (16.5)	
Have never used snuff	408 (51.8)	
Parenting experience	*n = *792	
First-time father	385 (48.6)	
Previous children	407 (51.4)	

a Several options could be chosen.

### Pregnancy planning and preconception lifestyle adjustments

Most of the pregnancies (81%, *n = *646) had been very or fairly planned, and 77% (*n = *601) of the fathers partly or fully agreed that it is mostly advantageous to plan a pregnancy (Table 3). A majority of the pregnancies (94%, *n = *738) had occurred spontaneously, i.e. without assisted reproductive technology. Median time to pregnancy was two months.

**Table 3.** Pregnancy-planning and preconception lifestyle adjustments in regard to the child born one year ago.

	Fathers, *n* (%)
How planned was the pregnancy?	*n* = 793
Very/fairly planned	646 (81.4)
Neither planned nor unplanned	79 (10.0)
Very/fairly unplanned	68 (8.6)
It is mostly advantageous to plan for a pregnancy	*n* = 785
Totally agree	332 (42.3)
Partly agree	269 (34.3)
No opinion	158 (20.2)
Partly disagree	22 (2.8)
Totally disagree	4 (0.5)
Mode of conception	*n* = 783
Spontaneous	738 (94.3)
Fertility treatment	45 (5.7)
Contraceptive use at the time of conception	*n* = 789
No	756 (95.8)
Yes, sometimes	21 (2.7)
Yes, but failed with usage	2 (0.3)
Yes, always	10 (1.3)
Right time to become parent?	*n* = 790
Yes	678 (85.8)
Not really, but it was OK	104 (13.2)
No	8 (1.0)
Preconception lifestyle adjustments^a^	*n* = 767
None	639 (83.3)
Reduced/quit smoking	26 (3.4)
Reduced/quit snuffing	27 (3.5)
Reduced/quit drinking alcohol	57 (7.4)
Reduced/quit drinking coffee	5 (0.7)
Exercised more	37 (4.8)
Exercised less	2 (0.3)
Adjusted medication	1 (0.1)
Ate healthier food	20 (2.6)
Sought medical/health advice	10 (1.3)
Other	6 (0.8)

a Several options could be chosen.

Most fathers (83%, *n* = 639) had not made any lifestyle adjustment prior to pregnancy to improve health and fertility. Among the fathers who had made an adjustment, 36% (*n = *46) had made more than one. Fathers who had made a lifestyle adjustment were on average 1.5 years younger than others (mean age 33.1 years and 34.6 years, *P* = 0.007). A total of 24% of first-time fathers had made a lifestyle adjustment, compared to 9% of fathers with previous children (*P* < 0.001). Lifestyle adjustment was more common if assisted reproductive technology (ART) had been used than if the pregnancy occurred spontaneously (43% versus 15%, *P* < 0.001). Median time to pregnancy was three months among men who had made an adjustment, which was one month longer than among the others (*P* = 0.018). There were no differences in terms of adjustment between men born in Sweden or abroad, men with high or low education, or in regard to level of pregnancy planning.

When looking at first-time fathers and experienced fathers separately, the patterns changed slightly. First-time fathers were more likely to make a lifestyle adjustment if they had used ART (*P* < 0.001), and the time to pregnancy was one month longer among those who had made an adjustment. In the group of experienced fathers, only age persisted as a significant variable. Men who had made an adjustment were two years younger than those who had not (mean age 33.9 years and 35.8 years, *P* = 0.038).

The most common adjustment was to reduce/quit the consumption of alcohol, cigarettes, or snuff, or to exercise more. Besides the predetermined options, 29 study participants described in free text which preconception lifestyle adjustment they had made. Most of the descriptions concerned changes in eating habits/diet, like reducing the intake of sugar, red meat, and fast food, or eating smaller portions. Fourteen men increased the intake of more wholesome food (vegetables, fruits and nuts, organic food) and/or began to take dietary supplements. One man wrote ‘Since my wife reduced her alcohol intake, I reduced mine as well’. As for other lifestyle adjustments, one man wrote that he had started running and another man that he had worn loose pants to optimize the environment for sperm production.

### Current family planning

Of the 789 men who answered the question about the wish for future children, 44% (*n = *347) wanted to have more children, 20% (*n = *158) were unsure, and 36% (*n = *284) did not want any more children. Most of the men who wanted more children (82%, *n = *285) only had one child at present. Half of them wanted to have their last child somewhere between the ages of 32 and 37 years, and it was most common to want in total two children; 15% (*n = *52) wanted to have their last child after the age of 40.

One out of four couples was not using any contraceptive method at the time being. Those who wanted more children were less likely to use a contraceptive method (60% usage) than those who were unsure (81% usage) or those who did not (88% usage, *P* < 0.001). Six percent (*n = *50) of the couples were currently expecting another child, and 25% of those pregnancies were estimated as very unplanned.

### Fertility knowledge

**Figure 2.**
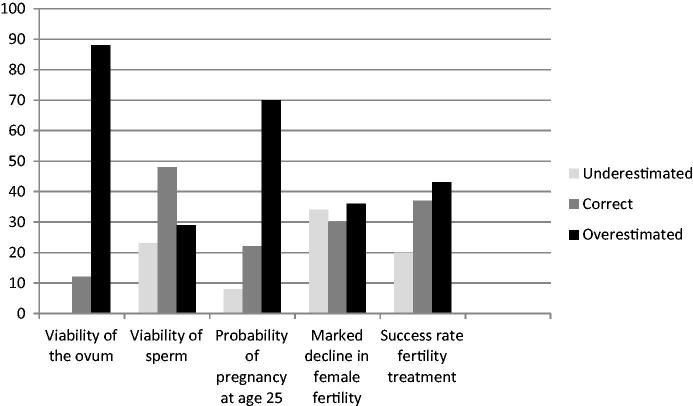
Percentages of underestimated, correct, and overestimated answers.

The range of answers to the fertility questions was wide (Table 4). The knowledge was highest on the issue of sperm viability; 48% were correct, and most of the other respondents were nearly correct. As for the other questions, the answers indicate a general overconfidence in fertility. The most common answer to the question about the viability of the ovum was that it is viable for three days. The probability for a woman to become pregnant during ovulation at the age of 25 was overestimated by 70% of the men. About one-third knew that a woman’s fertility markedly declines around the age of 35 years. The success rate of fertility treatment was on average slightly overestimated. Figure 2 gives an overview of the proportion of answers that were underestimates, correct, and overestimates.

**Table 4.** Fathers’ fertility knowledge. Median and interquartile range (IQR, 25th–75th percentile). Answers from five questions on fertility.

	Correct answer	Median (IQR)
1. How long is the ovum viable for fertilization?	0.5–1 day	3 days (2–5)
2. How long does sperm usually survive in the uterus/fallopian tubes?	2–3 days	2 days (2–4)
3. What is the probability that a 25-year-old woman becomes pregnant if she has unprotected intercourse with a young man at ovulation?	20%–30%	50% (30%–75%)
4. At what age begins a marked decline in a woman’s ability to conceive?	33–37 years	35 years (30–40)
5. What are the chances of having a child through IVF, for each attempt?	20%–30%	30% (20%–50%)

### Differences in fertility knowledge between groups with different socio-demographic characteristics

A larger proportion of fathers with higher education were knowledgeable of the success rate of fertility treatment than fathers with lower education (43% compared to 32%, *P* < 0.001). Highly educated men were also more often correct concerning the likelihood for a woman to become pregnant at 25 years (31% correct compared to 15%, *P* < 0.001). Fathers with lower education most often overestimated this likelihood. It was also more common among fathers with lower than higher education to overestimate at what age female fertility markedly declines (43% compared to 29%, *P* < 0.001).

More than half of fathers who had used fertility treatment (57%) were knowledgeable of the success rate of fertility treatment, in comparison to 36% of those who had experienced a spontaneous pregnancy (*P* = 0.021). There were no differences in fertility knowledge between fathers with different parental experience, country of birth, or level of pregnancy planning.

## Discussion

This study has provided insight into how a sample of recent fathers in Sweden plan and act before pregnancy. It was found that most pregnancies had been fairly or very planned, and that many participants considered planning as something advantageous. Lifestyle adjustments in preparation for pregnancy were not common practice but were, nevertheless, made by 17%. This could be regarded as a significant proportion considering that there are no official recommendations about preconception health directed to men in Sweden today. We interpret the prevalence of lifestyle adjustment as a sign of influence from a general health and lifestyle discourse and also as a means to support the pregnant woman. This will be further discussed below. Interestingly, men involved in planned pregnancies were not more prone to adjust their lifestyle nor had higher fertility knowledge than men involved in unplanned pregnancies. Hence, the question ‘How planned was the pregnancy?’ most likely reflects the level of intention to become a parent, but not the level of engagement in practical planning issues.

### Methodological considerations

This study includes a large sample of men, from a variety of urban and rural areas, occupations, income groups, and educational levels. It should, however, be noted that the sample is limited to fathers who understand Swedish. Consequently, the proportion of fathers with immigrant background (7.6%) is lower than the national average (∼20%). The sample also contains a greater proportion of fathers with a master’s or doctoral university degree, and very few were unemployed. When the longitudinal study started, 14% of the women stated that they had a partner born outside of Sweden, and 33.5% had a partner with university education. We conclude that many fathers with immigrant background were lost due to the fact that the questionnaire was only available in Swedish. Another difference from the general population is that more children were conceived with help from ART (5.7% compared to 3%). Finally, because of the study design, the results do not represent single fathers, fathers in same-sex relationships, or fathers with adopted children.

As for the recruitment of participants, we believe that antenatal care is a suitable place to recruit a representative sample of future parents since basically all pregnant women in Sweden attend antenatal care at some point in time (22). The recruitment of fathers in this study was, however, not made by personal contact but through the mothers, and at a late stage of a longitudinal study. Hence, the recruitment depended on the mother’s engagement, which makes the drop-out analysis very complex. We cannot calculate the exact response rate since we do not know exactly how many partners who received the invitation agreed to participate. The response rate of 41% (818 returned questionnaires out of 1,987 sent) is probably an underestimated figure, since the response rate of mothers was around 60%. If only the mothers who responded themselves invited their partner to take part, the response rate of partners would increase to 67%.

The recruitment and compliance could have been improved through personal contact with the eligible fathers-to-be at an earlier stage of the study, such as during an antenatal care visit. Still, recruitment of men to health surveys has previously been shown to be difficult. Tolonen et al. have described that, in Finland, response rates to health surveys have declined over the past 25 years, and the decline has been faster among men than among women (23). Also in the Swedish national survey ‘HIV in Sweden’, which was conducted every four years between 1987 and 2011, a significant decrease in response rate has been shown over the years, and men were less likely to respond than women (24).

Possible deficits from delaying the inclusion of fathers is the recall bias concerning pregnancy planning, and the lack of data on men’s lifestyles and health status before pregnancy. Concerning the first issue, we believe that most fathers can still remember one year after childbirth if the pregnancy was planned or not and if they changed their lifestyle, since a pregnancy is a significant life event. Also, the results are in line with the pilot study, where men were asked about pregnancy planning in early pregnancy (21). Concerning the second issue, we cannot identify men who would have benefited from lifestyle changes, such as weight loss. It is, however, likely that those who were obese or smoked at the time of data collection probably also were overweight and used tobacco at the time of conception. What we do not know is whether the fathers with previous children, who were less likely to make a lifestyle adjustment before pregnancy, actually had made a lifestyle adjustment before the conception of an older child, and if this can explain the difference.

### Preconception lifestyle

Our study is, to our knowledge, the first to describe preconception lifestyle adjustments among fathers who have not been recruited at a fertility clinic. The most common, but still rare, adjustment was a reduced intake of alcohol, which is interesting considering the limited evidence of alcohol’s negative impact on sperm quality. It is likely that some men reduced their intake to show support and solidarity with the woman, which was explicitly described by one participant and has also been presented as the most common motive for men to reduce alcohol consumption *during* pregnancy (25). Furthermore, most fathers who decreased their alcohol consumption also made one or several other lifestyle adjustments before pregnancy. This indicates a willingness to lead a healthier life in general and perhaps also to support the partner’s new lifestyle. According to Edvardsson et al., a healthy lifestyle during pregnancy is perceived by many parents as common knowledge, and since pregnancy is regarded as a shared responsibility the man’s role is meant to be equally important (26). Our results suggest that this view of equal responsibility extends into the pregnancy-planning stage.

In our study, reducing cigarette consumption was uncommon. The smokers were, however, few in number, also compared to national statistics of new fathers (27), which suggests that smoking has either been underreported or that the low number is related to the overrepresentation of highly educated men, who generally are less likely to be smokers. As for age, only 4% were older than 45 years, and most of the younger men wanted to have their last child before the age of 40. The age-related risk factor was thus also a minor issue.

### Fertility knowledge

Fertility knowledge in this study was similar to the knowledge of childless postgraduate male students in a previous Swedish study (7). It seems that high education is more important for the level of knowledge than having experience from a previous pregnancy, which we find particularly interesting. As theorized by Marsiglio et al., the process by which men become aware of their potential to procreate is relevant to their behaviour (17). Even though the majority of pregnancies in this study were planned, we figure that the procreative consciousness was not always raised or activated since most couples became pregnant within a rather short period of time. It is likely that few men experienced an incentive to learn more about fertility issues or to make a lifestyle adjustment.

### Implications for clinicians and policy-makers

Highlighting men’s preconception health is still a new phenomenon, both within research and within health care services. A recent review of preconception recommendations in six European countries reveals that preconception guidelines for men are generally missing (3), and several countries lack health services mandated to provide preconception counselling to men. Previous international studies suggest that many men of reproductive age are in need of family-planning and preconception care (28,29), but the intention among men to seek preconception care is low (30). However, we cannot expect men to seek preconception care if male reproductive health is not openly discussed, procreative consciousness stays low, and health care is inaccessible. The official objective of The National Board of Health and Welfare and The Public Health Agency of Sweden is ‘best possible sexual and reproductive health—on equal terms for the whole population and with fulfilment of everyone’s sexual and reproductive rights’. Still, today’s reproductive health care contributes to exclude men and put major responsibility on women, and thereby reproduce contemporary gendered norms for sexuality and reproduction. Similar gendered patterns have been found within Swedish child health care (31). This calls for a new societal approach. To improve both men’s and women’s reproductive health and decrease the risk of ill-health among offspring, there is work to be done on individual, group, and structural levels.

The trend that men with lower education had poorer knowledge is another inequality problem. We know from previous studies that reproductive treatment is mostly used by people with high socio-economic positions (32). Most probably, this means that low-resource couples with poor fertility knowledge and who have difficulties becoming pregnant will have poorer chances of becoming parents than others. This problem has to be dealt with on a structural and policy level. To promote equity in pregnancy outcomes, St Fleur et al. (33) recommend that future preconception care integrates evidence-based science about biomedical risks, toxic stress, and social determinants of health, with a transgenerational, epigenetic perspective.

### Unanswered questions and future research

From a public health perspective, it is relevant to highlight that more than 25% of the fathers were daily snuff (smokeless tobacco) users, a figure that is consistent with national statistics (34). Whether there is a relationship between snuff consumption and impaired male reproduction is still unclear, although there is evidence pointing in that direction (35). It is, however, known that smoking has negative effects on semen quality (36), and Swedish studies indicate that maternal snuff use in early pregnancy is associated with an increased risk of oral clefts in the child (37) and stillbirth (38).

## Conclusions

Our findings indicate that there is variation in how men plan and prepare for pregnancy. Both pregnancy-planning behaviour and fertility knowledge seem related to level of education and mode of conception. It is important to delve deeper into the meaning of the term ‘planned’ and what pregnancy planning entails according to men. An important problem, as we see it, is that men’s reproductive health and their need for preconception care is rarely discussed or problematized in society and health care. It is also necessary to assess which professional category could be suitable as provider of preconception care for men, and where and when the care could best be implemented. And not least, more research is needed on male fertility and lifestyle so that the advice to men is substantiated by evidence.
